# Discrimination of Urban Spaces with Different Level of Restorativeness Based on the Original and on a Shorter Version of Hartig et al.’s Perceived Restorativeness Scale

**DOI:** 10.3389/fpsyg.2017.01735

**Published:** 2017-10-09

**Authors:** Fátima Negrín, Estefanía Hernández-Fernaud, Stephany Hess, Bernardo Hernández

**Affiliations:** ^1^Psicología Cognitiva, Social y Organizacional, Universidad de La Laguna, San Cristóbal de La Laguna, Spain; ^2^Psicología Clínica, Psicobiología y Metodología, Universidad de La Laguna, San Cristóbal de La Laguna, Spain

**Keywords:** restorativeness, perceived restorativeness scale, shorter version, urban spaces, discrimination

## Abstract

Restorativeness is defined as the potential of the environment to re-establish certain cognitive capacities related to human information processing. The most frequently used instrument for evaluating the restorativeness of places is the Perceived Restorativeness Scale, proposed by Hartig et al. (1991). Later on, shorter versions of the Perceived Restorativeness Scale were proposed. The aim of this work is to evaluate the discriminatory capacity of the original and of a shorter Spanish version of the PRS, considering urban settings previously selected for having different level of restorativeness, according to expert’s criteria. The study involved 244 students and used a 3 × 2 mixed experimental design, with two independent variables: Restorativeness of a place (between-subjects), which was manipulated by showing pictures of settings selected with varying levels of restorativeness (high, medium, low), and length of the scale (within-subjects), which was manipulated by asking subjects to fill in both the original and a shorter version of the PRS. The order of presentation of the two scales was counterbalanced. Results show an appropriate reliability for both version of the scale. Items of being-away, fascination, and coherence of the shorter scale correlate more strongly with the corresponding factor of the original scale, compared to the others factors. Both scales produce similar values for the perceived restorativeness of the different places, except for places with low restorativeness.

## Introduction

For the past 30 years, there has been considerable interest in the effect of the physical environment on health and well-being (Milfont and Page, 2013). Empirical studies have shown that specific environmental features can help to reduce stress, improve attentional capacity, mood and general well-being, as well as physical and psychological rehabilitation, in both adults and children (Karjalainen et al., 2010; Velarde et al., 2010; Carrus et al., 2015). Specifically, restorativeness has been identified as a psycho-environmental process widely studied for its benefits to health and well-being. According to Hernández et al. (2001), restorativeness can be defined as the potential of specific settings of re-establishing certain cognitive capacities related to human information processing and executive functioning, particularly attention and concentration (Ulrich, 1981; Kaplan and Kaplan, 1989; Kaplan, 1995). Along these lines, Hartig (2010) defines the restorative capacity of the environment as the recovery of capacities and resources that are gradually depleted by the demands of everyday life.

Several authors have highlighted the importance of the restorative value of natural environments, including those located in urban settings (Purcell et al., 2001; Berto, 2005, 2007, 2014; Gulwadi, 2006; Velarde et al., 2010). For example, Purcell et al. (2001) found that environments that score higher in perceived restorativeness also score higher in terms of preference judgments, which appears to indicate that the perceived restorative value of an environment can be used as a framework for preference judgments. Similarly, the restorativeness of places has been found as an important correlate to positive affective bonds with places (Korpela and Hartig, 1996; Korpela et al., 2009), quality of life (Ruiz et al., 2013), stress reduction (Tyrväinen et al., 2014; Van den Berg et al., 2014), and sustainable behavior (Corral-Verdugo et al., 2012).

Kaplan (1995) describes restorative environments as those settings whose physical, spatial, and non-spatial features contribute to the recovery of psychological equilibrium. Considerable research in this field has focused on confirming the restorative effect of natural environments based on two theories, the Stress Recovery Theory (Ulrich, 1979, 1984) and the Attention Restoration Theory (Kaplan and Kaplan, 1989).

The Attention Restoration Theory sustains that the psychological benefits of nature are associated with the recovery of directed attention capacities. According to this approach, people use two types of attention: directed attention and fascination. Directed attention refers to the mental process required to handle cognitive data. It requires effort on the part of the individual and it is depleted after attentional efforts, when recovery is not possible. Directed attention can be recovered when the individual can make use of a second type of attention, fascination, which is generated spontaneously and effortlessly, and the prolonged use of which does not entail exhaustion.

Many everyday situations require directed attention in order to face them. This involves making an effort to direct attention so as to avoid any possible distraction and to recover that attention should it be lost for any reason. After these situations, the individual suffers what authors have termed directed attention fatigue or mental fatigue. Restorative natural surroundings trigger a fascination effect that restores the attentional capacity of the individual (Kaplan, 1995). This property of natural environments that reduces directed attention fatigue has been termed ‘restorativeness’ of places, occurring in ‘restorative environments’ (Hartig, 2004).

Kaplan and Kaplan (1989) propose four characteristics to identify restorative environments: being-away, fascination, extent, and compatibility. The presence of these four factors has been linked to beneficial effects on attentional recovery (Herzog et al., 2003). Being-away refers to the extent to which a place enables people to distance themselves from problems and daily concerns. Fascination is the capacity of the environment to effortlessly capture people’s attention. Extent refers to settings that feature space and coherence, thereby encouraging exploration of the surroundings. Compatibility is bound up with the extent to which a place fits people’s inclinations and interests. The measurement scales that have been developed are an attempt to contain these four, or often, five factors, given that the extent factor can also be divided into two: coherence and scope. Coherence is the property through which the different parts of a place are perceived as belonging to a whole. It represents the relationship between the content and structure of the setting, including aspects such as openness, mobility, and care for the surroundings. Scope refers to the perception of a setting as a place in which to enter and remain (Hartig et al., 1996; Pasini et al., 2009; Berto, 2014).

The procedure for measuring the restorativeness of place that has been used most frequently by research in this field is the subjective assessment of the restorative experience. Particular attention has been paid to the different components of perceived restorativeness, based on those described in the Attention Restoration Theory, outlined above (being-away, fascination, coherence, and compatibility).

Hartig and colleagues developed the first measure of perceived restorativeness, subsequently modified and published in 1996 and 1997 as the Perceived Restorativeness Scale (PRS; Hartig et al., 1996, 1997a,b). It is composed of 26 items initially organized as the four factors proposed in the Attention Restoration Theory, to which it was subsequently added a fifth factor, scope, referring to the extent to which the environment constitutes a ‘whole other world’ (Purcell et al., 2001). The scale has been translated into German (Hug et al., 2009) and Spanish, with its Spanish version being used in various Spanish-speaking countries, such as Spain itself (Hernández and Hidalgo, 2005) and Mexico (Martínez-Soto and Montero, 2010).

The adaptation of the PRS in Spain obtained a Cronbach’s alpha value of 0.93, while the values of the subscales were 0.88 for being-away, 0.67 for coherence, 0.86 for compatibility, 0.88 for fascination, and 0.61 for extent (Hernández and Hidalgo, 2005). These values are in line with those obtained in other studies (Korpela and Hartig, 1996; Purcell et al., 2001) and point to a good level of reliability, although the values of the factors coherence and scope can be improved.

Other instruments for measuring the restorativeness of place were also proposed later on, which include: (1) the Restorative Components of Environments Scale (Laumann et al., 2001), composed of 22 items that result in a structure of five factors, with a factor labeled as “Novelty” being added to the previous scales; (2) the Perceived Restorative Characteristics Questionnaire, also based on the same five factors, and developed to specifically evaluate the perceived restorativeness of zoo attractions (Pals et al., 2009), and (3) the Restorative State Scale developed to evaluate the change in restorative state (Van den Berg et al., 2014). A specific scale for use with children, the Perceived Restorative Components Scale for Children, has also been developed (Bagot et al., 2007) and adapted to Spanish by Corraliza et al. (2012).

Shorter versions of the PRS have also been used, in an attempt to simplify the measurement and have a more practical instrument: for example, a 16-item version of the original scale was proposed by Hartig and colleagues themselves (RPRS; Hartig et al., 1997b); a 17-item Self-rating Restoration Scale (RS) was used by Han (2003); and an 8-item version of the PRS was proposed by Berto (2005). Berto’s (2005) proposal is composed by one item for each factor of the Korpela and Hartig’s (1996) Perceived Restorativeness Scale, resulting in a five-item scale. This version was also used by Carrus et al. (2013), who obtained a Cronbach’s alpha of 0.87 for the entire scale and showed its capacity to discriminate between places with different degrees of naturalness. More recently, Carrus et al. (2017) also used this short version to measure the perceived restorativeness of botanical gardens in Italy. Ruiz and Hernández (2014) developed a Spanish version of this shorter form, and used it in a study carried out in the Canary Islands, during a volcanic eruption on the island of El Hierro and following a forest fire in the island of La Gomera (Hidalgo et al., 2013), obtaining Cronbach’s alphas higher than 0.80.

Shorter versions of scales with discriminatory capacities similar to longer measures are useful both in research contexts and for applied purposes. The objective of this study is to analyze the discriminatory capacity of the original and a shorter version of the perceived restorativeness scale in Spanish language. We base our main hypothesis on the capacity of the shorter version of the perceived restorativeness scale to discriminate between urban spaces with different levels of natural elements in the same way as the original scale.

## Materials and Methods

### Participants

The sample was composed of 244 second- and third-year psychology undergraduates from the University of La Laguna (Tenerife, Spain), of whom 54 were men (22.13%) and 190 (77.87%) women. The mean age was 20.55 years, with a standard deviation of 3.81 years.

### Design

A 3 × 2 mixed experimental design was used, with two independent variables: one between-subject, and one within-subject. The between-subject variable was the restorativeness level of the place, and was manipulated by asking subject to assess pictures of places that were pre-selected for likely having different levels of restorativeness (high, medium, and low). The within-subject variable was the length of the scale, and was manipulated by asking subject to fill both the original and a shorter version of the Perceived Restorativeness Scale. The order of presentation of the two scales was counterbalanced across the whole sample.

### Materials and Tools

We used photographic material as stimuli for the manipulation of the restorativeness of the place variable. We took 120 photographs of 24 different places located in the Campus de Guajara, of the University of La Laguna, in Tenerife, Spain. Pictures were taken on a sunny day and from a variety of angles. In the first phase, three expert judges rejected 42 photographs that did not meet the appropriate conditions of luminosity, clarity, and perspective of place. The judges who evaluated the photographs had experience in psycho-environmental research and extensive knowledge in photographic techniques.

**FIGURE 1:**
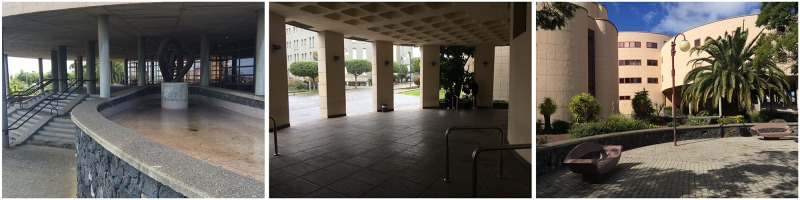
Photographs of places with a low level of restorativeness.

**FIGURE 2:**
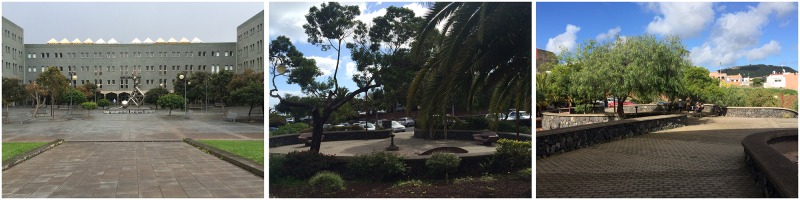
Photographs of places with a medium level of restorativeness.

**FIGURE 3:**
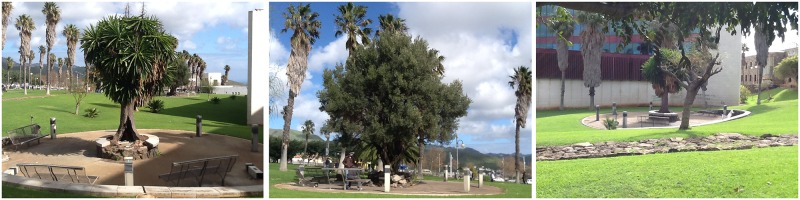
Photographs of places with a high level of restorativeness.

The judges subsequently applied the Delphi method to evaluate the remaining pictures. In this second phase, the pictures were classified according to three levels of restorativeness (low, medium, and high), on the basis of their naturalness (e.g., Grahn and Stigsdotter, 2009). When there was no agreement among the judges, the picture was deleted. Thus, 31 pictures were deleted. Finally, the 47 remaining pictures were reanalyzed to select the more prototypical pictures for each level of restorativeness. Again, when an image did not receive a unanimous evaluation from all the judges, it was deleted. This process was repeated until three pictures were finally selected for each of the three levels of restorativeness. Finally, nine pictures were then chosen: three for each place with low, medium, and high levels of restorativeness, based on the presence of natural elements such as trees and grassy areas (see **Figures 1–3**). A similar procedure has been successfully employed by other authors in previous studies (Grahn and Stigsdotter, 2009; Nordh et al., 2009, 2010).

As stated previously, our questionnaire included two instruments to measure perceived restorativeness: the Spanish version of the original Hartig et al.’s (1996) Perceived Restorativeness Scale (adapted to Spanish by Hernández and Hidalgo, 2005) and a Spanish short version of the same scale, proposed by Ruiz and Hernández (2014). Socio-demographic data were also recorded. Each scale is described below in more detail.

*Perceived Restorativeness Scale*: we used the version adapted to Spanish by Hernández and Hidalgo (2005), which includes 26 items that assess the extent to which the setting helps people forget the stresses and strains of everyday life, feel relaxed, move around freely, keep curiosity alive, and avoid boredom. These items were grouped into five factors: being-away, fascination, compatibility, coherence, and scope. The response scale ranges from 0, which means “Not at all,” to 10, which means “Totally.” It also includes two items that assess the extent to which the place is attractive to people and the extent to which they prefer this place to others.

*Short version in Spanish of the Perceived Restorativeness Scale* (Ruiz and Hernández, 2014): this scale is composed of the following five items: “This place lets me forget my everyday responsibilities, feel relaxed, and lose myself in my own thoughts”; “This is a fascinating place that keeps my curiosity alive and stops me from getting bored”; “This is a place where activities and things are orderly and well organized”; “This place is another world, where I can move around at ease”; and “I feel comfortable here because it’s easy to find your way around this place,” which correspond to the factors of being-way, fascination, coherence, compatibility, and scope, respectively. The response scale ranges from 0, which means “Not at all,” to 10, which means “Totally.”

We devised two version of the questionnaire, in order to counterbalance the order of presentation of the two scales (long and short version).

### Procedure

Each photograph was assessed by a different group of students in a group session. They were required to answer the questionnaire in reference to the photo shown. Mean response time was 20 min during which the image was always projected. The questionnaire was given in pencil and paper format, and was completed individually and anonymously.

The participants completed the questionnaire during a practical class of the degree in Psychology. Participation was voluntary. Participants did not get credits or monetary compensation for collaborating.

### Ethics Statement

Because the study involved no risk to participants, informed consent was given verbally. Participants were clearly informed that the participation was voluntary and that there would be no compensation for participation. The University of La Laguna Ethics Committee in Tenerife, Spain (ULLECT) approved this study. All relevant data are available via the Harvard Dataverse at https://dataverse.harvard.edu/dataset.xhtml?persistentId=doi:10.7910/DVN/YM2NT7

### Statistical Analysis

First of all, the absence of multivariate outliers was tested using Mahalanobis distance. Secondly, the structures of the scales were tested using confirmatory factor analysis. Thirdly, reliability was calculated for the total score of each perceived restorativeness scale, and for the single factors of the original scale. Fourthly, the score for each factor of the Perceived Restorativeness Scale was calculated by the linear combination of the items belonging to each one. Fifthly, differences between mean scores were analyzed using an ANOVA to check for any effect of the order of presentation of the scales and to compare the scores for each of the three level of restorativeness manipulated through the picture presentation. Sixthly, descriptive statistics and correlations between the two scales were calculated. Finally, a repeated measure analysis of variance was performed. The confidence level was set to 95%, so a *p*-value ≤ 0.05 was considered significant.

## Results

The structures of the scales were tested using confirmatory factor analysis. For the original PRS, five first order factors and one second order factor was confirmed [χ^2^(293) = 797.612, BNFI = 0.933, BNNFI = 0.952, CFI = 0.956, RMSEA = 0.087, RMSEA confidence interval = 0.080,0.094]. For the short version of the PRS, a unidimensional structure was confirmed [χ^2^(5) = 38.579, BNFI = 0.930, BNNFI = 0.934, CFI = 0.942, RMSEA = 0.169, RMSEA confidence interval = 0.121,0.219].

Reliability was calculated for the total of each perceived restorativeness scale, with a Cronbach’s alpha of 0.92 and 0.79, for the original and short versions, respectively. Internal consistency of the factors of the original scale was high for being-away (0.85), fascination (0.69), coherence (0.86), and compatibility (0.80), and low for scope (0.46).

No significant differences were found in the mean scores on the basis of the order of presentation of the PRS either in its original [*t*(242) = -0.746; *p* = 0.456; η^2^= 0.002) or short version [*t*(242) = -1.570; *p* = 0.118; η^2^= 0.01). Likewise, no statistically significant differences were found in the overall mean scores obtained with the original Perceived Restorativeness Scale among the pictures within each level of restorativeness: high level [*F*(2,79) = 0.668; *p* < 0.515; η^2^= 0.017], medium level [*F*(2,84) = 0.148; *p* < 0.862], and low level [*F*(2,72) = 2.704; *p* < 0.074; η^2^= 0.07]. Thus, the pictures representing the three places for each of the three level of restorativeness can be considered as homogenous and were coded as places with a high, medium, and low level of restorativeness.

**Table 1** shows the descriptive statistics of restorativeness, pleasure, and preference for each level of restorativeness and for the entire sample. As expected, higher scores were obtained for the perceived restorativeness of places with a higher level of restorativeness, with both the original and short versions of the scale. Likewise, places with a high level of restorativeness received higher scores in terms of pleasure and preference.

**Table 1:** Descriptive statistics of Perceived Restorativeness (original and short versions of the scale), Pleasure, and Preference for each level of restorativeness of place.

	Original PRS		Short version		Pleasure		Preference	
Restorative level	*M*	*SD*	*M*	*SD*	*M*	*SD*	*M*	*SD*
Low (*n* = 75)	3.90	1.49	3.59	1.74	4.85	2.61	1.38	2.09
Medium (*n* = 87)	4.50	1.40	4.42	1.50	5.96	2.40	2.87	2.45
High (*n* = 82)	5.18	1.48	5.40	1.79	7.05	2.28	5.12	2.99
Total	4.54	1.54	4.50	1.82	5.95	2.59	3.17	2.95


Bivariate correlations were calculated among perceived restorativeness scores measured through both scales, pleasure, and preference, for each level of restorativeness and for the entire sample (**Table 2**). As shown in **Table 2**, high correlations were detected between the two scales of perceived restorativeness, both when considering the total sample and also when considering each of the levels of restorativeness separately. Moreover, moderate and high correlations were obtained between the two PRS scores and the pleasure and preference scores.

**Table 2:** Correlations between Perceived Restorativeness (original and short version of the scale), Pleasure, and Preference for each level of restorativeness of places and for the entire sample.

Restorative level	Perceived restorativeness scale	Short version	Pleasure	Preference
Low	Original	0.86^∗^	0.71^∗^	0.51^∗^
	Short		0.57^∗^	0.43^∗^
Medium	Original	0.78^∗^	0.64^∗^	0.49^∗^
	Short		0.58^∗^	0.51^∗^
High	Original	0.73^∗^	0.70^∗^	0.41^∗^
	Short		0.67^∗^	0.50^∗^
Total	Original	0.81^∗^	0.72^∗^	0.54^∗^
	Short		0.65^∗^	0.58^∗^


^∗^*p < 0.01*.

We calculated the correlation of the components of the original scale with the items of the short version for the total sample and for each level of restorativeness. For the total sample, we obtained significant correlations, high or moderate, between each component of the original scale and the corresponding item of the short version of the scale (see **Table 3**). We also obtained significant and positive correlations between each component of the original scale and the other items in the short version of the scale. As displayed in **Table 3**, in particular the items of compatibility and scope in the short version of the scale correlate highly with factors of the original scale other than the theoretically equivalent factor.

**Table 3:** Correlation between the five factors of the original scale and the items of the short version according to the restorative level of place and for the entire sample.

		Short version of the scale				
Restorative level	Original scale factor	Being-away item	Fascination item	Coherence item	Compatibility item	Scope item
Low	Being-away	** 0.66^∗∗^**	0.62^∗∗^	0.29^∗^	0.45^∗∗^	0.44^∗∗^
	Fascination	0.33^∗∗^	** 0.72^∗∗^**	0.49^∗∗^	0.65^∗∗^	0.52^∗∗^
	Coherence	0.16	0.28^∗^	** 0.34^∗∗^**	0.22	0.59^∗∗^
	Compatibility	0.26^∗^	0.62^∗∗^	0.34^∗∗^	** 0.61^∗∗^**	0.73^∗∗^
	Scope	0.23	0.56^∗∗^	0.30^∗^	0.64^∗∗^	** 0.58^∗∗^**
Medium	Being-away	** 0.70^∗∗^**	0.46^∗∗^	0.14	0.16	0.33^∗∗^
	Fascination	0.35^∗∗^	** 0.61^∗∗^**	0.25^∗^	0.54^∗∗^	0.49^∗∗^
	Coherence	0.18	0.21^∗^	** 0.58^∗∗^**	0.38^∗∗^	0.46^∗∗^
	Compatibility	0.32^∗∗^	0.45^∗∗^	0.25^∗^	** 0.52^∗∗^**	0.52^∗∗^
	Scope	0.24^∗^	0.39^∗∗^	0.20	0.65^∗∗^	** 0.51^∗∗^**
High	Being-away	** 0.67^∗∗^**	0.32^∗∗^	0.27^∗^	0.41^∗∗^	0.47^∗∗^
	Fascination	0.43^∗∗^	** 0.65^∗∗^**	0.53^∗∗^	0.54^∗∗^	0.47^∗∗^
	Coherence	0.20	0.31^∗∗^	** 0.58^∗∗^**	0.29^∗^	0.49^∗∗^
	Compatibility	0.45^∗∗^	0.42^∗∗^	0.31^∗∗^	** 0.49^∗∗^**	0.48^∗∗^
	Scope	0.46^∗∗^	0.41^∗∗^	0.34^∗∗^	0.49^∗∗^	** 0.46^∗∗^**
Total	Being-away	** 0.72^∗∗^**	0.52^∗∗^	0.26^∗∗^	0.41^∗∗^	0.45^∗∗^
	Fascination	0.44^∗∗^	** 0.69^∗∗^**	0.44^∗∗^	0.62^∗∗^	0.53^∗∗^
	Coherence	0.20^∗∗^	0.29^∗∗^	** 0.50^∗∗^**	0.31^∗∗^	0.52^∗∗^
	Compatibility	0.42^∗∗^	0.55^∗∗^	0.33^∗∗^	** 0.59^∗∗^**	0.62^∗∗^
	Scope	0.36^∗∗^	0.48^∗∗^	0.29^∗∗^	0.60^∗∗^	** 0.53^∗∗^**

^∗^*p < 0.05; ^∗∗^p < 0.01*. The values in bold are the correlations between each factor of the original scale and its corresponding item of the shorter scale.

In the same way as for the entire sample, the correlation between the factors of the original scale and their corresponding item in the short version is the highest in each level of restorativeness for being-away, fascination, and coherence, except in the case the low level of restorativeness. In compatibility and scope, high correlations are also observed between the items of the short version and factors other than the corresponding theoretical item of the long version of the scale (**Table 3**).

**FIGURE 4:**
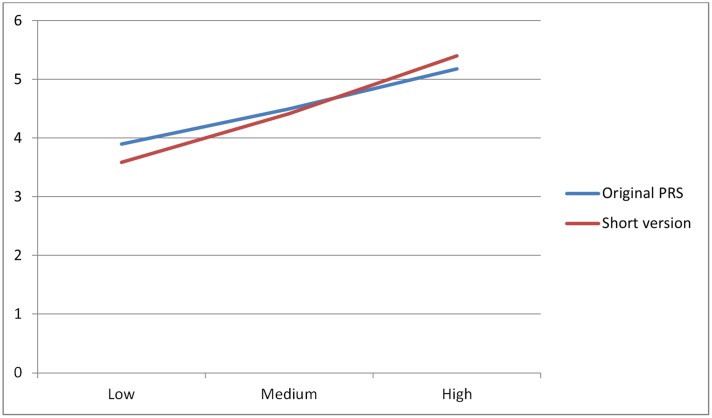
Representation of the effect of the interaction between Level of restorativeness of place and Scale.

Finally, we performed repeated measure ANOVA, where the between-subjects factor was restorativeness of the place, with three levels (low, medium, and high), and the within-subject factor was length of the scale, with two levels (original and short version of the scale). The multivariate homogeneity of variances was checked against the M-Box test, which was not significant. The two-way interaction was statistically significant [*F*(2,241) = 4.94; *p* = 0.008; η2 = 0.04]. Moreover, the main effect of the between-subjects factor (restorativeness of the place) was significant: [*F*(2,241) = 21.44, *p* = 0.000; η^2^= 0.15]. The within-subjects factor (length of the scale) did not have statistically significant effect. Only in the case of pictures representing places with low level of restorativeness we detected significant differences between the scores obtained with the shorter (*M* = 3.58; *SD* = 1.75) vs. the original version of the perceived restorativeness scale (*M* = 3.89; *SD* = 1.49) [*t*(74) = 2.94, *p* = 0.012; see **Figure 4**].

## Discussion

Restorativeness refers to the characteristics of places that facilitate recovery from the depletion of cognitive and attentional resources (Ulrich, 1981; Kaplan and Kaplan, 1989; Kaplan, 1995; Hernández et al., 2001; Hartig, 2010). Environments with natural elements possess this property, producing a positive effect on quality of life, reducing stress, establishing affective bonds with places or psychological well-being in general (Ulrich, 1983; Ulrich et al., 1991; Korpela et al., 2009; Ruiz et al., 2013; Van den Berg et al., 2014; Carrus et al., 2015).

The development of instruments to measure the perceived restorativeness of environments has been the object of important research in this field. To that end, several scales have been created, the most utilized and researched being the Perceived Restorativeness Scale (Hartig et al., 1996, 1997a,b), of which several shorter versions have been proposed. The objective of this study is to analyze the capacity of the Perceived Restorativeness Scale, in its original and a shorter version of five items, to discriminate between urban settings with high and low levels of restorativeness, in a Spanish context. Given the increasingly multi-cultural and multilingual character of current human societies (e.g., Pirchio et al., 2017), the possibility of having different linguistic version of widely used instruments in psychological studies such as the Hartig et al. (1996) Perceived Restorativeness Scale, is also an important aim for environmental psychological research.

The results obtained show that the two scales present good internal consistency and have a high positive correlation, both for the entire sample and for each group of participants in each of the levels of restorativeness analyzed. The value of the internal consistency of the scales is in line with those obtained by Purcell et al. (2001) and Berto (2007) for the original scale, and by Berto (2005) and White et al. (2010) for shorter versions of the perceived restorativeness scale. Both the original and short versions of the scale correlate positively and significantly with pleasure and preference for place. This result is in line with previous findings from several authors, showing positive correlations between the perceived restorativeness and the preference (Laumann et al., 2001; Purcell et al., 2001; Herzog et al., 2003; Berto, 2007; Tenngart Ivarsson and Hagerhall, 2008; Nordh et al., 2009; Pals et al., 2009).

The correlation of each item of the short version with the equivalent factor of the original version was higher (compared to correlations between the single item and the non-corrensponding factors), in the case of being-away, fascination, and coherence, for the total sample and for the medium and high levels of restorativeness (only in the case of the factor fascination a correlation higher with the coherence single item was detected, but limited to the assessment of pictures on the low level of restorativeness). This is not the case with the items of compatibility and scope of the short version, which present higher correlations with factors other than their equivalent factor in the original scale. Overall, these results might also suggest the possibility of a one-dimensional structure of the perceived restorativeness construct. Although the original scale enables the differentiation between the different theoretical factors, in fact, perceived restorativeness might represent a single theoretical construct when measured through shorter instruments.

Finally, both scales allow to discriminate between places with a low, medium, and high level of restorativeness, with no statistically significant differences between the scores obtained with each of them, except in the low level of restorativeness, in which the short version gives a lower mean value than the original scale. This study also allows to compare a longer vs. shorter version of the PRS through a within-subject design, obtaining results that are in line with those obtained in studies with different samples for the original and short versions (Purcell et al., 2001; Berto, 2005, 2007; Velarde et al., 2010). It is interesting to point out that in previous studies, urban settings combining several natural elements, as is the case of the stimuli used in this study, were found to differ in their restorative potential (Scopelliti and Giuliani, 2004; Tenngart Ivarsson and Hagerhall, 2008). Both the original perceived restorativeness scale and the short version of five items used here are sensitive to these differences. In this regard, the short version of the scale, when compared with the original, gives lower mean values of restorativeness for places selected for having less restorative properties, and values that tend to be higher for settings with more restorative properties. These results could indicate that the short version has a greater capacity to discriminate between urban places with different levels of restorative elements, even when the mean scores in the low and high levels of restorativeness are not extreme scores on the scale, or that the short version might lead to overestimate differences in the restorative potential of different places. In future research, it would be interesting to check this fact by comparing places with even greater differences in their objective restorativeness, in order to test for even stronger discrimination capacity, and also to assess the relations between perceived restorativeness and other related constructs, such as mindfulness or pro-environmental behavior (e.g., Lymeus et al., 2016; Panno et al., 2017).

This research has some limitations that must be considered because they may affect the generalizability of the results. The use of a students sample is a limitation, because it does not represent the general population. Also, although it is usual in this type of studies, to use pictures as stimuli may have reduced the overall perceived restorativeness judgements. Finally, the participants very likely had a previous knowledge of the places represented in the pictures used as stimuli, since they were all located in the university campus. Although previous contact or familiarity with the places may have influenced the results, both the two versions of the PRS used here have recorded this effect.

## Conclusion

This work shows that both the original and a shorter Spanish version of the perceived restorativeness scale have similar characteristics, and have the power to discriminate between urban settings with different levels of restorative properties. The usefulness of the short version of the scale, in addition to the advantages of having a brief instrument for research, lies in the availability of an instrument that is easier to implement in applied population studies that normally are more comfortably reached through shorter measuring instruments.

## Author Contributions

FN: acquisition, analysis, and interpretation of data, drafting the work, final approval of the version to be under-reviewed. EH-F: conception and design of the work, analysis and interpretation of data, drafting the work and revising it critically for important intellectual content, final approval of the version to be under-reviewed. SH: analysis and interpretation of data, final approval of the version to be under-reviewed. BH: conception and design of the work, analysis and interpretation of data, drafting the work and revising it critically for important intellectual content, final approval of the version to be under-reviewed.

## Conflict of Interest Statement

The authors declare that the research was conducted in the absence of any commercial or financial relationships that could be construed as a potential conflict of interest. The reviewer AP and handling Editor declared their shared affiliation, and the handling Editor states that the process nevertheless met the standards of a fair and objective review.

## References

[B1] Bagot K. L. Kuo F. E. Allen F. C. L. (2007). Amendments to the Perceived Restorative Components Scale for Children (PRCS-C II). *Child. Youth Environ.* 17 124–127.

[B2] Berto R. (2005). Exposure to restorative environments helps restore attentional capacity. *J. Environ. Psychol.* 25 249–259. 10.1016/j.jenvp.2005.07.001

[B3] Berto R. (2007). Assessing the restorative value of the environment: a study on the elderly in comparison with young adults and adolescents. *Int. J. Psychol.* 42 331–341. 10.1080/00207590601000590

[B4] Berto R. (2014). The role of nature in coping with psycho-physiological stress: a literature review on restorativeness. *Behav. Sci.* 4 394–409. 10.3390/bs4040392 25431444 PMC4287696

[B5] Carrus G. Lafortezza R. Colangelo G. Dentamaro I. Scopelliti M. Sanesi G. (2013). Relations between naturalness and perceived restorativeness of different urban green. *Psyecology* 4 227–244. 10.1174/217119713807749869

[B6] Carrus G. Passiatore Y. Pirchio S. Scopelliti M. (2015). Contact with nature in educational settings might help cognitive functioning and promote positive social behaviour. *Psyecology* 6 191–212.

[B7] Carrus G. Scopelliti M. Panno A. Lafortezza R. Colangelo G. Pirchio S. (2017). A different way to stay in touch with ‘urban nature’: the perceived restorative qualities of botanical gardens. *Front. Psychol.* 8:914 10.3389/fpsyg.2017.00914 PMC5450850 28620335

[B8] Corraliza J. A. Collado S. Bethelmy L. (2012). Children’s perceived restoration: adaptation of the PRCS for children to a spanish sample. *Psyecology* 3 195–204. 10.1174/217119712800337729

[B9] Corral-Verdugo V. Tapia C. García F. Varela C. Cuen A. Barrón M. (2012). Validation of a scale assessing psychological restoration associated with sustainable behaviours. *Psyecology* 3 87–100. 10.1174/217119712799240242

[B10] Grahn P. Stigsdotter U. K. (2009). The relation between perceived sensory dimensions of urban green space and stress restoration. *Landsc. Urban Plan.* 94 264–275. 10.1016/j.lamdurbplan.2009.10.012

[B11] Gulwadi G. B. (2006). Seeking restorative experiences. Elementary school teachers′ choices for places that enable coping with stress. *Environ. Behav.* 4 503–520. 10.1177/0013916505283420

[B12] Han K. T. (2003). A reliable and valid self-rating measure of the restorative quality of natural environments. *Landsc. Urban Plan.* 64 209–232. 10.1016/s0169-2046(02)00241-4

[B13] Hartig T. (2004). “Restorative environments,” in *Encyclopedia of Applied Psychology* Vol. 3 ed. Spielberger C. (San Diego, CA: Academic Press), 273–279.

[B14] Hartig T. (2010). “Issues in restorative environments research: matters of measurement,” in *Psicología Ambiental 2011: Entre los Estudios Urbanos y el Análisis de la Sostenibilidad*, eds Fernández-Ramírez B. Hidalgo C. Salvador C. M. Martos M. J. (Almería: Universidad de Almería y Asociación de Psicología Ambiental), 41–66.

[B15] Hartig T. Korpela K. Evans G. W. Garling T. (1997a). A measure of restorative quality in environments. *Scand. J. Psychol.* 37 378–393. 10.1080/02815739708730435

[B16] Hartig T. Korpela K. Evans G. W. Garling T. (1997b). A measure of perceived environmental restorativeness. *Scand. Hous. Plan. Res.* 14 175–194. 10.1080/02815739708730435

[B17] Hartig T. Korpela K. M. Evans G. W. Garling T. (1996). *Validation of a Measure of Perceived Environmental Restorativeness. (Göteborg Psychological Reports*, 26:7). Göteborg: Göteborg University.

[B18] Hartig T. Mang M. Evans G. W. (1991). Restorative effects of natural environments experiences. *Environ. Behav.* 23 3–26. 10.1177/0013916591231001

[B19] Hernández B. Hidalgo M. C. (2005). Effect of urban vegetation on psychological restorativeness. *Psychol. Rep.* 96 1025–1028. 10.2466/pr0.96.3c.1025-1028 16173374

[B20] Hernández B. Hidalgo M. C. Berto R. Perón E. (2001). The role of familiarity on the restorative value of a place. Research on a Spanish sample. *Bull. People Environ. Stud.* 18 22–25.

[B21] Herzog T. R. Maguire C. P. Nebel M. B. (2003). Assessing the restorative components of environments. *J. Environ. Psychol.* 23 159–170. 10.1016/s0272-4944(02)00113-5

[B22] Hidalgo M. Hernández B. Ruiz C. Negrín F. (2013). “*¿Es el lugar de apego un lugar restaurador?*,” in *Proceedings of the XI Congreso Psicología Ambiental*. Barcelona.

[B23] Hug S. Hartig T. Hansmann R. Seeland K. Hornung R. (2009). Restorative qualities of indoor and outdoor exercise settings as predictors of exercise frequency. *Health Place* 15 971–980. 10.1016/j.healthplace.2009.03.002 19427807

[B24] Kaplan R. Kaplan S. (1989). *The Experience of Nature. A Psychological Perspective.* Cambridge: Cambridge University Press.

[B25] Kaplan S. (1995). The restorative benefits of nature: toward an integrative framework. *J. Environ. Psychol.* 15 169–182. 10.1016/0272-4944(95)90001-2

[B26] Karjalainen E. Sarjala T. Raitio H. (2010). Promoting human health through forests: overview and major challenges. *Environ. Health Prev. Med.* 15 1–8. 10.1007/s12199-008-0069-2 19568838 PMC2793342

[B27] Korpela K. Ylén M. Tyrväinen L. Silvennoinen H. (2009). Stability of self-reported favourite places and place attachment over a 10-month period. *J. Environ. Psychol.* 29 95–100. 10.1016/j.jenvp.2008.05.008

[B28] Korpela M. K. Hartig T. (1996). Restorative qualities of favorite places. *J. Environ. Psychol.* 12 249–258. 10.1006/jevp.1996.0018

[B29] Laumann K. Gärling T. Stormark K. (2001). Rating scale measures of restorative components of environments. *J. Environ. Psychol.* 21 31–44. 10.1006/jevp.2000.0179

[B30] Lymeus F. Lundgren T. Hartig T. (2016). Attentional effort of beginning mindfulness training is offset with practice directed toward images of natural scenery. *Environ. Behav.* 49 536–559. 10.1177/0013916516657390

[B31] Martínez-Soto J. Montero M. (2010). Percepción de cualidades restauradoras y preferencia ambiental. *Rev. Mex. Psicol.* 27 183–190.

[B32] Milfont T. L. Page E. (2013). A bibliometric review of the first thirty years of the journal of environmental psychology. *Psyecology* 4 195–216. 10.1174/217119713806144366

[B33] Nordh H. Hagerhall C. M. Holmqvist K. (2010). Exploring view pattern and analyzing pupil size as a measure of restorative qualities in park photos. *Acta Hortic.* 881 767–772. 10.17660/ActaHortic.2010.881.126

[B34] Nordh H. Hartig T. Hagerhall C. M. Fry G. (2009). Components of small urban parks that predict the possibility for restoration. *Urban For. Urban Green.* 8 225–235. 10.1016/j.ufug.2009.06.003

[B35] Pals R. Steg L. Siero F. W. van der Zee K. I. (2009). Development of the PRCQ: a measure of perceived restorative characteristics of zoo attractions. *J. Environ. Psychol.* 29 441–449. 10.1016/j.jenvp.2009.08.005

[B36] Panno A. Giacomantonio M. Carrus G. Maricchiolo F. Pirchio S. Mannetti L. (2017). Mindfulness, pro-environmental behavior, and belief in climate change: the mediating role of social dominance. *Environ. Behav.* 10.1177/0013916517718887 [Epub ahead of print].

[B37] Pasini M. Berto R. Scopelliti M. Carrus G. (2009). Measuring the restorative qualities of the environments: contribution to the validation of the Italian version of the Perceived Restorativeness Scale. *Boll. Psicol. Appl.* 257 3–11.

[B38] Pirchio S. Passiatore Y. Carrus G. Maricchiolo F. Taeschner T. Arcidiacono F. (2017). Teachers and parents involvement for a good school experience of native and immigrant children. *J. Educ. Cult. Psychol. Stud.* 15 73–94. 10.7358/ecps-2017-015-pirc

[B39] Purcell T. Peron E. Berto R. (2001). Why do preferences differ between scene types? *Environ. Behav.* 33 93–106. 10.1177/00139160121972882

[B40] Ruiz C. Hernández B. (2014). Emotions and coping strategies during an episode of volcanic activity and their relations to place attachment. *J. Environ. Psychol.* 38 279–287. 10.1016/j.jenvp.2014.03.008

[B41] Ruiz C. Pérez C. Hernández B. (2013). Apego al lugar, restauración percibida y calidad de vida: un modelo de relación. *Estud. Psicol.* 34 315–321. 10.1174/021093913808349271

[B42] Scopelliti M. Giuliani M. V. (2004). Choosing restorative environments across the lifespan: a matter of place experience. *J. Environ. Psychol.* 24 423–437. 10.1016/j.jenvp.2004.11.002

[B43] Tenngart Ivarsson C. Hagerhall C. M. (2008). The perceived restorativeness of gardens – Assessing the restorativeness of a mixed built and natural scene type. *Urban For. Urban Green.* 7 107–118. 10.1016/j.ufug.2008.01.001

[B44] Tyrväinen L. Ojala A. Korpela K. Lanki T. Tsunetsugu Y. Kagawa T. (2014). The influence of urban green environments on stress relief measures: a field experiment. *J. Environ. Psychol.* 38 1–9. 10.1016/j.jenvp.2013.12.005

[B45] Ulrich R. S. (1979). Visual landscapes and psychological well-being. *Landsc. Res.* 4 17–23. 10.1080/01426397908705892

[B46] Ulrich R. S. (1981). Natural versus urban scenes: some psychophysiological effects. *Environ. Behav.* 13 523–556. 10.1177/0013916581135001

[B47] Ulrich R. S. (1983). “Aesthetic and affective response to natural environment,” in *Behavior and the Natural Environment* Vol. 6 eds Altman I. Wohlwill J. F. (New York, NY: Plenum Press), 85–125.

[B48] Ulrich R. S. (1984). View through a window may influence recovery from surgery. *Science* 224 420–421. 10.1126/science.6143402 6143402

[B49] Ulrich R. S. Simons R. F. Losito B. D. Fiorito E. Miles M. A. Zelson M. (1991). Stress recovery during exposure to natural and urban environments. *J. Environ. Psychol.* 11 201–230. 10.1016/s0272-4944(05)80184-7

[B50] Van den Berg A. E. Jorgensen A. Wilson E. R. (2014). Evaluating restoration in urban green spaces: does setting type make a difference? *Landsc. Urban Plan.* 127 173–181. 10.1016/j.landurbplan.2014.04.012

[B51] Velarde M. D. Tveit M. S. Hagerhall C. M. (2010). “The link between lanscape preferences and perceived restorativeness. Current research trends and suggestions for future studies,” in *Environmental Psychology: New Developments*, eds Valentín J. Gámez L. (New York, NY: Nova Science Publishers), 235–242.

[B52] White M. Smith A. Humphryes K. Pahl S. Snelling D. Depledge M. (2010). Blue space: the importance of water for preference, affect, and restorativeness ratings of natural and built scenes. *J. Environ. Psychol.* 30 482–493. 10.1016/j.jenvp.2010.04.004

